# Analysis of the codon usage pattern of the RdRP gene of mycovirus infecting *Aspergillus* spp.

**DOI:** 10.1186/s12985-019-1115-y

**Published:** 2019-01-16

**Authors:** Mikyung Je, Hayeon Kim, Hyeon S. Son

**Affiliations:** 1 SNU Bioinformatics Institute, Interdisciplinary Graduate Program in Bioinformatics, College of Natural Science, Seoul National University, 1 Gwanak-ro, Gwanak-gu, Seoul 08826 Korea; 2 Laboratory of Computational Biology & Bioinformatics, Institute of Public Health and Environment, Graduate School of Public Health, Seoul National University, 1 Gwanak-ro, Gwanak-gu, Seoul 08826 Korea; 3 Department of Biomedical Laboratory Science, Kyungdong University, 815 Gyeonhwon-ro, Munmak, Wonju, Gangwondo 24695 Korea

**Keywords:** Mycovirus, Polymycovirus, AfuPmV-1, Codon usage pattern, RSCU

## Abstract

**Background:**

Mycoviruses that infect fungi generally do not have a significant effect on the host and, instead, reduce the toxicity of the fungi. However, recent studies have shown that polymycovirus-1, a mycovirus that infects *Aspergillus* species known to cause disease in humans, is related to increased virulence of the fungus.

**Methods:**

Comparative analysis was performed of RdRP gene codon usage patterns of *Aspergillus fumigatus* polymycovirus-1 (AfuPmV-1) and other mycoviruses known to infect *Aspergillus* spp. to examine the genetic characteristics of AfuPmV-1. In addition, codon usage analysis was performed to determine whether the nucleotide composition and codon usage characteristics of AfuPmV-1 were also present in other polymycoviruses and hypervirulence-related mycoviruses. Phylogenetic analysis was also performed to investigate their evolutionary relationship.

**Results:**

Analysis of nucleotide composition indicated that AfuPmV-1 had the highest GC content among analyzed mycoviruses and relative synonymous codon usage analysis indicated that all of the codons preferred by AfuPmV-1 ended with C or G, while codons ending with A or U were not observed. Moreover, the effective number of codons, the codon adaptation index, and correspondence analysis showed that AfuPmV-1 had greater codon preference compared with other mycoviruses and that AfuPmV-1 had relatively high adaptability to humans and fungi. These results were generally similar among polymycoviruses.

**Conclusions:**

The codon usage pattern of AfuPmV-1 differs from other mycoviruses that infect *Aspergillus* spp. This difference may be related to the hypervirulence effect of AfuPmV-1. Analysis of AfuPmV-1 codon usage patterns could contribute to the identification and prediction of virulence effects of mycoviruses with similar genetic characteristics.

## Background

Mycoviruses are viruses that infect fungi and are known to be infectious to most fungal species. While mycoviruses have little or no influence on the fungal host in most cases, some have been shown to control the pathogenicity of the host by increasing or decreasing the virulence [1, 2]. To date, research on mycoviruses has mainly focused on plant pathogenic fungi. The hypovirulence effect of mycoviruses in fungi has led to the development of biological control agents to reduce the virulence of fungi on important crops and plant resources [2, 3]. There have been relatively few studies on the hypervirulence effect of mycoviruses, although some results have been reported recently. In 2015, the A78 virus, which infects the human pathogenic fungus, *Aspergillus fumigatus*, was reported to have significant mild hypervirulence effects in the moth species, *Galleria mellonella* [4], and similar virulence effects have been reported in *Aspergillus fumigatus* tetramycovirus-1 (AfuTmV-1) found in the same fungus [5]. AfuTmV-1 was renamed AfuPmV-1, and its virus family name was changed from *Tetramycoviridae* to *Polymycoviridae* as more viruses showing similar characteristics to AfuTmV-1 were discovered, including BbPmV-1, which infects the insect pathogenic fungus, *Beauveria bassiana* [6] (Table 1).

**Table 1:** The genome of polymycovirus-1 (AfuTmV-1)

Host	Segment (bp)	ORF		Product	
		Gene	Length	Protein	Length (aa)
*Aspergillus fumigatus*	dsRNA1 (2403)	RdRP ORF	2292 nt	RNA dependent RNA polymerase	763
	dsRNA2 (2233)	unknown_ORF	2091 nt	hypothetical protein	696
	dsRNA3 (1970)	Methyl transferase ORF	1845 nt	methyl transferase	614
	dsRNA4 (1131)	PAS-rp ORF	840 nt	proline/alanine/serine (PAS)-rich protein	279

*Aspergillus* is a fungus that belongs to the phylum *Ascomycota* and is related to various human diseases through infection, including aspergillosis on infection of the lungs, asthma, and allergies. Moreover, it is one of the fatal human pathogenic fungi, with a high mortality rate in patients with low immunity by opportunistic infection [7, 8]. Although studies have focused on polymycovirus-1 with regard to the increase in fungal pathogenicity in *A. fumigatus*, it is also important to examine other mycoviruses, such as partitivirus, chrysovirus, and victorivirus, which are infectious to other species in the genus *Aspergillus*, including *A. fumigatus*, *A. foetidus*, *A. ochraceus*, and *A. niger*. This study was performed to explore other mycoviruses that may increase the pathogenicity of the genus *Aspergillus* by comparison with polymycovirus-1, and to investigate the genetic characteristics of mycoviruses that enhance the toxicity of human pathogenic fungi.

## Methods

### Sequence data collection

The AfuPmV-1 genome consists of four capped double-stranded RNAs (dsRNAs), of which the largest dsRNA1 segment encodes the RNA-dependent RNA polymerase (RdRP) [5, 6]. The data were downloaded from the NCBI GenBank database (https://www.ncbi.nlm.nih.gov/genbank) and the complete nucleotide sequences of the RdRP coding region of mycoviruses that infect *Aspergillus* species were used. Eight sets of data published between 2006 and 2015 were studied in the analysis. Other polymycoviruses and hypervirulence-related mycoviruses data were downloaded from NCBI for comparison (Table 2).

**Table 2:** Summary of datasets

No.	Accession no.		Fungal host	Mycovirus	Abbr.	Length		Ref.
	Nucleotide	Protein				Nucleotide (nt)	Protein (aa)	
1	FN376847	CAY25801	*Aspergillus fumigatus*	partitivirus-1	AfuPV-1	1629	542	[22]
2	FN178512	CAX48749	*Aspergillus fumigatus*	chrysovirus	AfuCV	3345	1114	[23]
3	HG975302	CDP74618	*Aspergillus fumigatus*	polymycovirus-1^b^	AfuPmV-1	2292	763	[5]
4	HE588144	CCD33020	*Aspergillus foetidus*	alternavirus^c^	AfAltV	3375	1124	[24]
5	HE588147	CCD33024	*Aspergillus foetidus*	victorivirus^d^	AfVV	2520	839	[25]
6	HE588148	CCD33025	*Aspergillus foetidus*	exartavirus^e^	AfExV	2889	962	[26]
7	DQ270031	ABC86749	*Aspergillus ochraceus*	partitivirus	AoPV	1620	539	[27]
8	EU289897^a^	ABX79997	*Aspergillus niger*	alternavirus	AniAltV	3375	1124	[28]
9	MF444217	AWY10945	*Sclerotinia sclerotiorum*	tetramycovirus-1	SstRV1	2403	800	[29]
10	MF317878	AVZ65983	*Penicillium digitatum*	polymycoviruses 1	PdPmV1	2283	760	[30]
11	LN896307	CUS18595	*Beauveria bassiana*	polymycovirus 1	BbPmV-1	2328	775	[6]
12	LN896311	CUS18599	*Beauveria bassiana*	polymycovirus 2	BbPmV-2	2304	767	[6]
13	LC350277	BBC27878	*Alternaria alternata*	chrysovirus 1	AaCV1	3354	1174	[31]
14	KM235317	AKF14154	*Penicillium marneffei*	partitivirus-1	TmPV1	1638	545	[32]

No. 1~8: mycoviruses infecting *Aspergillus* (Group 1); No. 9~12: other polymycoviruses (Group 2); No. 13~14: hypervirulence-related mycoviruses (Group 3)

^a^putative RdRP gene

^b^originally named *Aspergillus fumigatus* tetramycovirus-1

^c^proposed name of *Aspergillus foetidu*s fast virus

^d^proposed name of *Aspergillus foetidus* slow virus 1

^e^proposed name of *Aspergillus foetidus* slow virus 2

### Codon usage analysis

The programs CodonW (https://sourceforge.net/projects/codonw/) and CALcal (http://genomes.urv.es/CAIcal/) were used to conduct analyses of nucleotide composition, overall/local G + C content, relative synonymous codon usage (RSCU), effective number of codons (ENC), codon adaptation index (CAI), and correspondence analysis (COA) for each of the selected genetic data [9, 10]. Nucleotide composition analysis was conducted based on the overall frequency of nucleic acid occurrence (A%, C%, U%, and G%), total AU%/GC%, frequency of the third nucleic acid in synonymous codons (A3s, C3s, U3 s, and G3 s), and the GC3s values. The RSCU is a value represented as the ratio between predicted and observed usage rates of specific codons, assuming that all synonymous codons were used equally for an amino acid. An RSCU value of 1.0 indicates that all codons were used randomly or equally. The value would be greater (less) than 1.0 if certain codons were used more (less) frequently [11, 12]. In particular, codons with RSCU values ≥1.6 are considered over-represented codons, while those with RSCU values ≤0.6 are considered under-represented codons [13, 14]. The ENC is a simple and absolute method to measure codon usage bias in genes and genomes. The GC3s values are used in the calculation, and the ENC value ranges from 20 to 61. The ENC is inversely proportional to codon usage bias, and lower ENC values indicate stronger codon usage bias. A gene that does not show any codon usage bias would have an ENC value of 61. On the other hand, a gene that shows a high degree of codon usage bias would have an ENC value of 20, indicating that only one codon was used for an amino acid. Generally, ENC values < 35 are interpreted as showing strong codon usage bias [15, 16]. The CAI is the geometric mean of relative adaptation level and is a quantitative method to calculate the differences in codon usage bias against highly expressed known reference data. The CAI value ranges between 0 and 1, with values closer to 1 indicating a high degree of similarity between the reference data and the codon usage pattern and expression level, whereas smaller values indicate lower similarity in the codon usage pattern and expression level [17]. The reference data of codon usage levels in humans (*Homo sapiens*) and fungus (*Aspergillus fumigatus*) were acquired from the codon usage database (http://www.kazusa.or.jp/codon/) for comparison [18]. Furthermore, the RSCU values were examined by the COA method with the XLSTAT 2016 program for visualization of the CodonW results. The COA method is the preferred method in the area of multivariate analysis and represents the data as vectors consisting of rows and columns [19]. Each individual data of the RdRP codon region was represented as a vector with 59 dimensions covering 59 codons except methionine (AUG) and tryptophan (UGG), which lack synonymous codons, as well as termination codons. A comparative analysis was performed including three groups: mycoviruses infecting *Aspergillus* (Group 1), polymycoviruses with a fungal host other than *Aspergillus* (Group 2), and newly reported mycoviruses that enhance host virulence (Group 3).

### Phylogenetic analysis

The phylogenetic relationships among the mycoviruses used for the analysis were inspected and phylogenetic analysis was conducted using the MEGA7 program (http://www.megasoftware.net) to infer the influence of evolutionary processes on codon usage patterns. RdRP coding sequences were aligned with the MUSCLE algorithm, and the phylogenetic tree was constructed by applying the Maximum Likelihood method and the Kimura 2-parameter substitution model. The robustness of the tree was verified with the bootstrap value set to 1000 [20].

## Results

### Nucleotide composition features

**Fig. 1:**
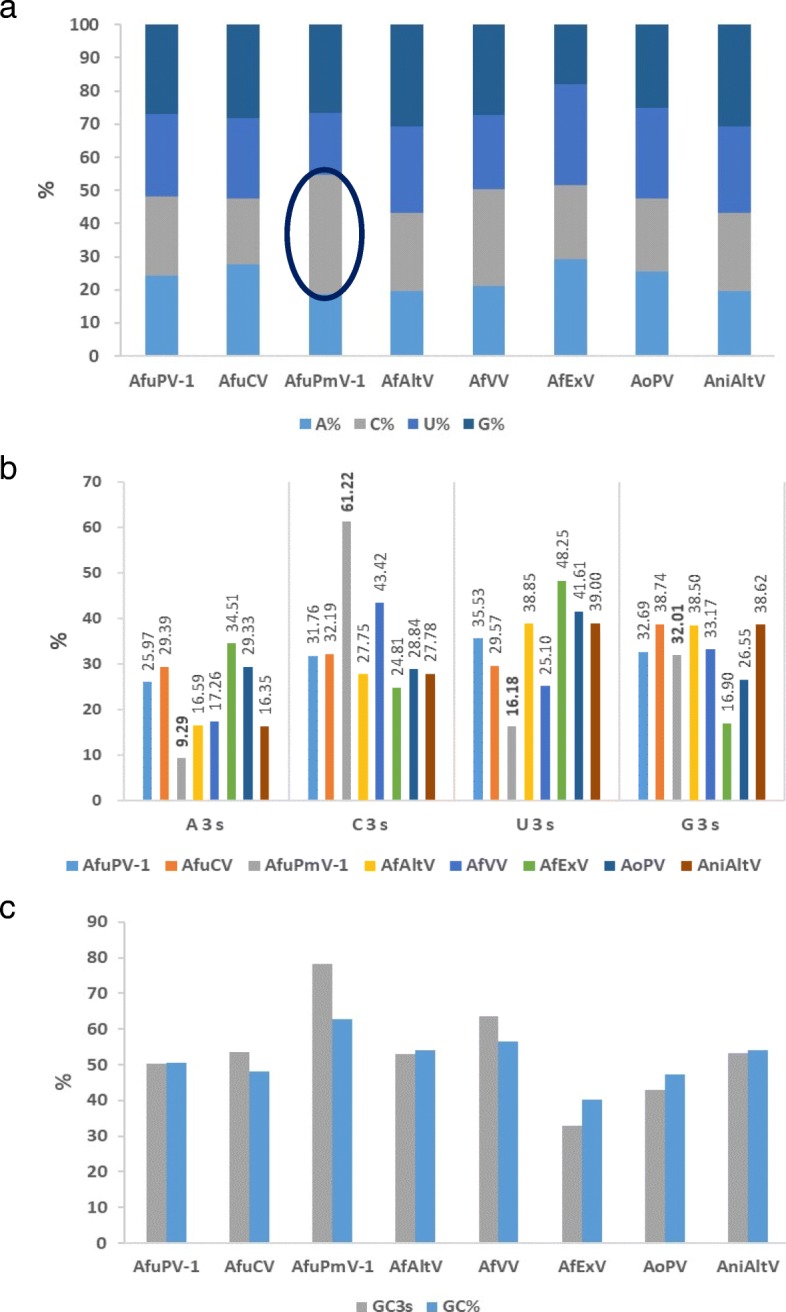
Nucleotide composition features. **a** Nucleotide distribution of A, C, U, and G in the RNA-dependent RNA polymerase (RdRp) gene. **b** Nucleotide distribution frequency calculated only for the third codon base. **c** Analysis of GC content at all codon positions (GC%) and at the third position (GC3s)

Basic nucleotide composition analysis was conducted for the RdRP gene of mycoviruses infecting *Aspergillus* spp. (Table 3, Fig. 1). The mean and standard deviation (SD) of A%, C%, U%, and G% were 23.16 ± 4.00, 25.09 ± 5.13, 25.13 ± 3.54, and 26.63 ± 4.04, respectively. The mean and SD of total AU% and GC% were 48.29 ± 6.76 and 51.71 ± 6.76, respectively. The mean and SD of A3s, C3s, U3 s, G3 s, and GC3s were 22.34 ± 8.66, 34.72 ± 12.09, 34.26 ± 10.20, 32.15 ± 7.47, and 53.48 ± 13.45, respectively. AfuPmV-1 showed the lowest frequencies of nucleotides A and U and the highest frequency of nucleotide C among the *Aspergillus*-infecting mycoviruses included in the analysis. Similarly, the frequencies of the four types of nucleotides in the third position of synonymous codons were the lowest with A3s and U3 s and highest with C3s. As nucleotide C occurred relatively abundantly in AfuPmV-1, the GC content and GC3s indices were highest in this virus with values of 62.74 and 78.30, respectively (Fig. 1). The GC content of AfuPmV-1 was reported to be approximately 63% in a previous study of the entire or partial genome sequences of *Polymycoviridae* viruses [6]. As a result of the comparison, Group 2 showed a similar pattern to that of AfuPmV-1. The GC content was between 59.97 and 61.98%, and the frequencies of nucleotides at the third position in the synonymous codons were higher in C3s and G3 s than in A3s and U3 s, respectively. Among these, BbPmV-1 is an experimentally reported virus that may be related to mild hypervirulence. The Group 3 results differed from those of Groups 1 and 2 (Table 3).

**Table 3:**
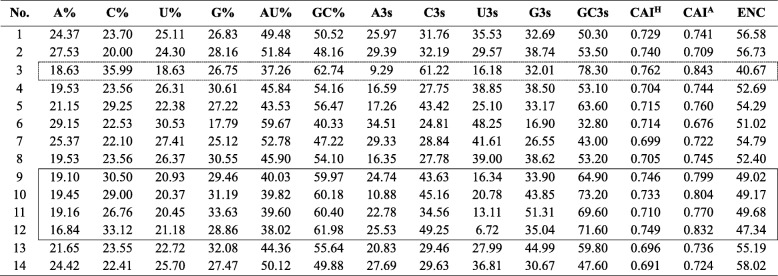
Nucleotide compositions of the RdRP genes of *Aspergillus*-infecting mycoviruses

CAI^H^: compare with *Homo sapiens* as reference set

CAI^A^: compare with *Aspergillus fumigatus* as reference set

The dotted line shows the result of AfuPmV-1 analysis, and the solid line shows the result of analysis of polymycoviruses

### RSCU value and codon usage preference

**Fig. 2:**
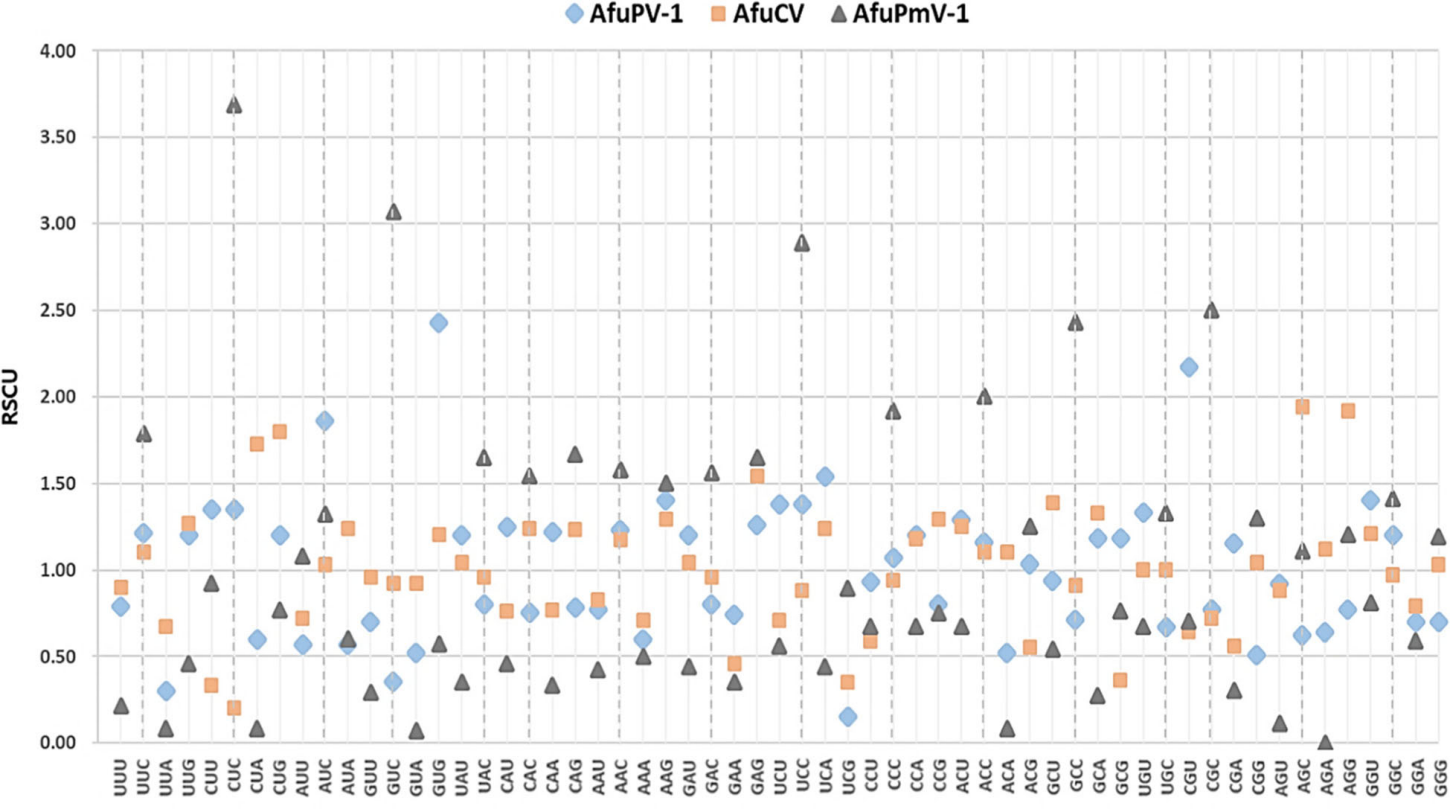
Relative synonymous codon usage (RSCU) analysis of AfuPmV-1, AfuCV, and AfuPV-1. C-ended codons were the most preferred in AfuPmV-1 and AfuPmV-1 had no A/U-ended codon in codons with an RSCU value of 1.0 or higher (gray dotted line: C-ended codon)

The RSCU values of AfuPmV-1, AfuCV, and AfuPV-1 of Group1 were compared to inspect the codon usage bias according to virus species (Table 4, Fig. 2). Of codons related to the entire 18 amino acids, 18 codons were preferred in AfuPmv-1 of which 11 showed RSCU values ≥1.6. AfuCV showed 17 preferred codons, three of which had RSCU values ≥1.6. AfuPV had 20 preferred codons, three of which showed RSCU values ≥1.6. AfuPmV-1 showed similarities to AfuCV in codons CAC (His), CAG (Gln) and to AfuPV-1 in codons CUC (Leu) and AUC (Ile). The codons preferred by each virus individually were as follows. The codons solely preferred in AfuPmV-1 were GUC (Val), UAC (Tyr), GAC (Asp), UCC (Ser), CCC (Pro), ACC (Thr), GCC (Ala), UGC (Cys), CGC (Arg), and GGC (Gly) for 10 of the amino acids. Remarkably, all of these were C-ended codons (Fig. 2). Codons solely preferred in AfuCV were CUG (Leu), AUA (Ile), CCG (Pro), GCU (Ala), AGC (Ser), and AGG (Arg) for six of the amino acids, and in AfuPV-1, CUU (Leu), CAU (His), CAA (Gln), UCA (Ser), CCA (Pro), GCA (Ala), GCG (Ala), UGU (Cys), and CGU (Arg) for eight of the amino acids. The codons UUC (Phe), AAC (Asn), AAG (Lys), and GAG (Glu) showed similar preferences in all three viruses. The RSCU values and end nucleotide composition indicated that, in the RdRP coding region, C-ended codons were strongly preferred in AfuPmV-1 (15 of 18), G-ended codons were preferred in AfuCV (7 of 17), and U-ended codons were preferred in AfuPV-1 (8 of 20). Interestingly, there were no A/U-ended codons among the preferred codons of AfuPmV-1, indicating that AfuPmV-1 has a codon bias toward C- and G-ended codons.

**Table 4:** RSCU analysis of AfuPmV-1, AfuCV, and AfuPV-1

AA	Codon	AfuPmV-1	AfuCV	AfuPV-1	AA	Codon	AfuPmV-1	AfuCV	AfuPV-1
Phe	UUU	0.21^a^	0.90	0.79	Ser	UCU	0.56^a^	0.71	1.38
	UUC	**1.79** ^b^	**1.10**	**1.21**		UCC	**2.89** ^b^	0.88	1.38
Leu	UUA	0.08^a^	0.67	0.30		UCA	0.44^a^	1.24	**1.54**
	UUG	0.46^a^	1.27	1.20		UCG	0.89	0.35^a^	0.15^a^
	CUU	0.92	0.33^a^	**1.35**	Pro	CCU	0.67	0.59^a^	0.93
	CUC	**3.69** ^b^	0.20^a^	**1.35**		CCC	**1.92** ^b^	0.94	1.07
	CUA	0.08^a^	1.73	0.60^a^		CCA	0.67	1.18	**1.20**
	CUG	0.77	**1.80** ^b^	1.20		CCG	0.75	**1.29**	0.80
Ile	AUU	1.08	0.72	0.57^a^	Thr	ACU	0.67	**1.25**	**1.29**
	AUC	**1.32**	1.03	**1.86** ^b^		ACC	**2.00** ^b^	1.10	1.16
	AUA	0.60^a^	**1.24**	0.57^a^		ACA	0.08^a^	1.10	0.52^a^
Val	GUU	0.29^a^	0.96	0.70		ACG	1.25	0.55^a^	1.03
	GUC	**3.07** ^b^	0.92	0.35^a^	Ala	GCU	0.54^a^	**1.39**	0.94
	GUA	0.07^a^	0.92	0.52^a^		GCC	**2.43** ^b^	0.91	0.71
	GUG	0.57^a^	**1.20**	**2.43** ^b^		GCA	0.27^a^	1.33	**1.18**
Tyr	UAU	0.35^a^	**1.04**	**1.20**		GCG	0.76	0.36^a^	**1.18**
	UAC	**1.65** ^b^	0.96	0.80	Cys	UGU	0.67	1.00	**1.33**
His	CAU	0.46^a^	0.76	**1.25**		UGC	**1.33**	1.00	0.67
	CAC	**1.54**	**1.24**	0.75	Arg	CGU	0.70	0.64	**2.17** ^b^
Gln	CAA	0.33^a^	0.77	**1.22**		CGC	**2.50** ^b^	0.72	0.77
	CAG	**1.67** ^b^	**1.23**	0.78		CGA	0.30^a^	0.56^a^	1.15
Asn	AAU	0.42^a^	0.83	0.77		CGG	1.30	1.04	0.51^a^
	AAC	**1.58**	**1.17**	**1.23**	Ser	AGU	0.11^a^	0.88	0.92
Lys	AAA	0.50^a^	0.71	0.60^a^		AGC	1.11	**1.94** ^b^	0.62
	AAG	**1.50**	**1.29**	**1.40**	Arg	AGA	0.00^a^	1.12	0.64
Asp	GAU	0.44^a^	**1.04**	**1.20**		AGG	1.20	**1.92** ^b^	0.77
	GAC	**1.56**	0.96	0.80	Gly	GGU	0.81	**1.21**	**1.40**
Glu	GAA	0.35^a^	0.46^a^	0.74		GGC	**1.41**	0.97	1.20
	GAG	**1.65** ^b^	**1.54**	**1.26**		GGA	0.59^a^	0.79	0.70
						GGG	1.19	1.03	0.70

*AA* amino acid

Non-degenerate amino acids (Met, Trp) and stop codons are excluded

The boldface are the most preferred codons

^a^RSCU value ≤0.6; under-represented codons

^b^RSCU value ≥1.6; over-represented codons

Group 2 showed a similar codon usage pattern to that of AfuPmV-1, which indicates a preference for the C/G-ended codon. Group 3 also preferred C- or G-ended codons, but the four nucleic acids were distributed more evenly.

### General codon usage pattern

**Fig. 3:**
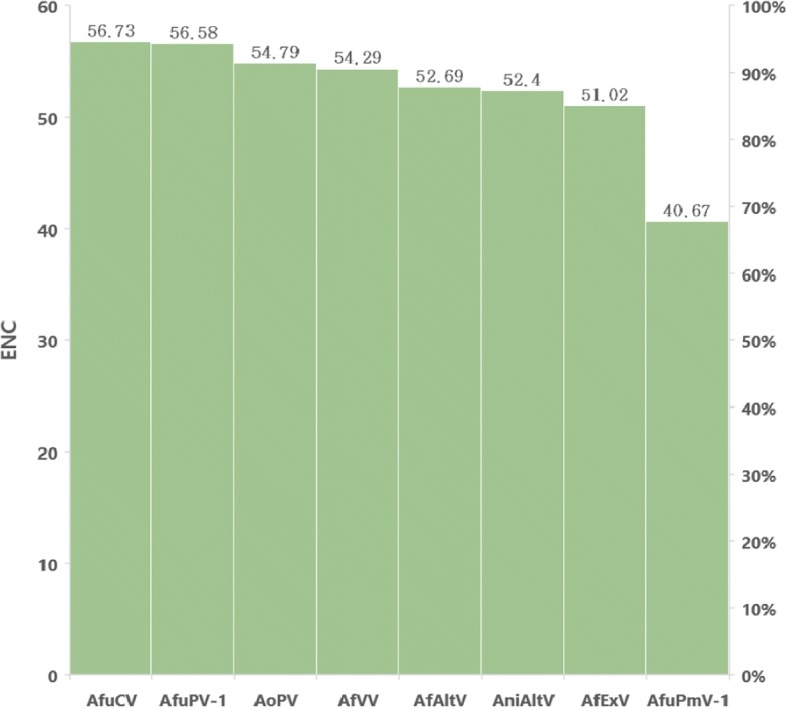
Effective number of codons (ENC) value in the eight mycoviruses infecting Aspergillus. The average ENC value was 52.40 and the ENC value of AfuPmV-1 was 40.67. The low ENC value of AfuPmV-1 means that it has strong codon usage bias compared to other mycoviruses

The ENC value was calculated to quantitatively measure the magnitude of RdRP gene codon usage bias of the eight mycoviruses infecting *Aspergillus* spp. An ENC value < 35 indicates a strong codon usage bias. The results showed that the ENC value was lowest for AfuPmV-1 (40.67) and the other viruses showed ENC values > 50 (Table 3, Fig. 3). Taking into account the results of previous studies on RNA viruses in which the ENC values of Zaire Ebola virus, Chikungunya virus, Hepatitis C virus, and West Nile virus were 57.23, 55.56, 52.62, and 53.81, respectively [21], AfuPmV-1 appeared to have stronger codon usage bias relative to other viruses. The CAI was calculated to compare the adaptability of synonymous genetic codon usage in mutually different individuals, and the codon usage patterns were considered similar to the reference individual with CAI values closer to 1. This study referred to the CAI values of *H. sapiens* and *A. fumigatus*, which have ranges of 0.699–0.762 and 0.676–0.843, respectively. The mean and SD were 0.72 ± 0.02 and 0.74 ± 0.05 for *H. sapiens* and *A. fumigatus*, respectively. Remarkably, AfuPmV-1 showed the highest values for both *H. sapiens* and *A. fumigatus* with values of 0.762 and 0.843, respectively. These results indicated that AfuPmV-1 has the greatest similarity to the reference data in codon usage pattern and expression level, and that it has higher adaptability to human and fungal hosts compared with other mycoviruses. In Group 2, CAI and ENC values were similar to those of AfuPmV-1 (Table 3).

### General trend of codon usage variation

**Fig. 4:**
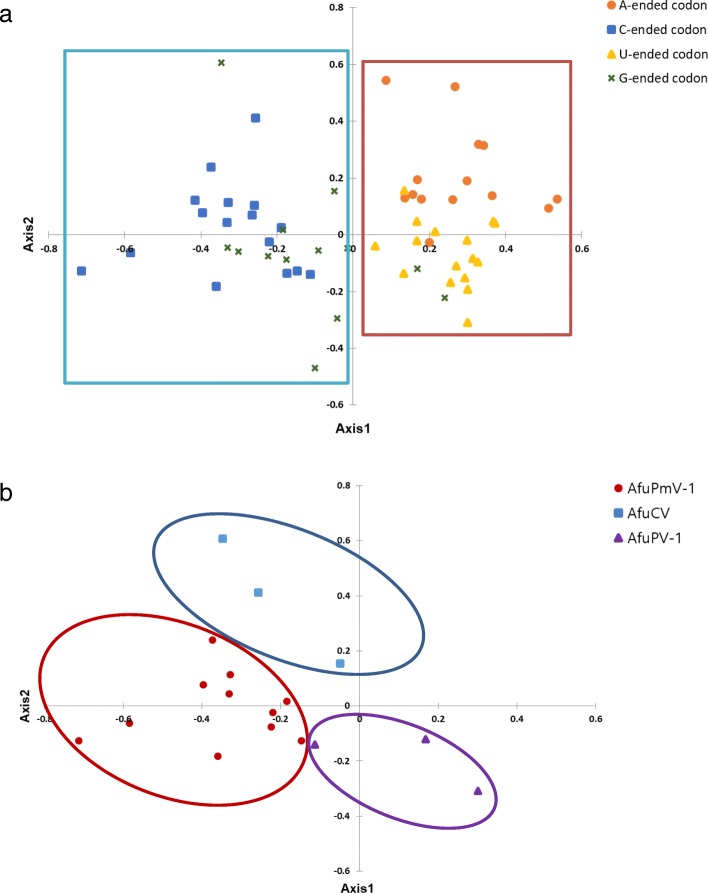
Correspondence analysis results using RSCU values (COA-RSCU). Axis1 and Axis2 of the COA-RSCU explained 44.31 and 18.81%, respectively, of the total variation. **a** COA result for A/C/U/G-ended codons; G- and C-ended codons formed one group and A- and U-ended codons formed one group. **b** COA result for over-represented codons (RSCU > 1.6); the codons strongly preferred individually by the three viruses formed separate groups

To inspect the trends related to codon usage patterns of the *Aspergillus*-infecting viruses AfuPmV-1, AfuCV, and AfuPV-1, COA was performed with the RSCU values. Axis1, Axis2, Axis3, and Axis4 of the COA-RSCU explained 44.31, 18.81, 17.33, and 9.78% of the total variation, respectively. The results were based on 59 codons, excluding the three termination codons and methionine (AUG)/tryptophan (UGG) that do not have synonymous codons. Although there were exceptions in the COA results according to the third nucleotide in the codon, G- and C-ended codons formed one group and A- and U-ended codons formed another group (Fig. 4(a)). Moreover, the COA results for over-represented codons with RSCU values ≥1.6 showed that the codons strongly preferred individually by the three viruses formed separate groups (Fig. 4(b)). The observations verified that there were differences in the codon pattern preferences among the *Aspergillus*-infecting viruses.

### Evolutionary relationship between mycoviruses

**Fig. 5:**
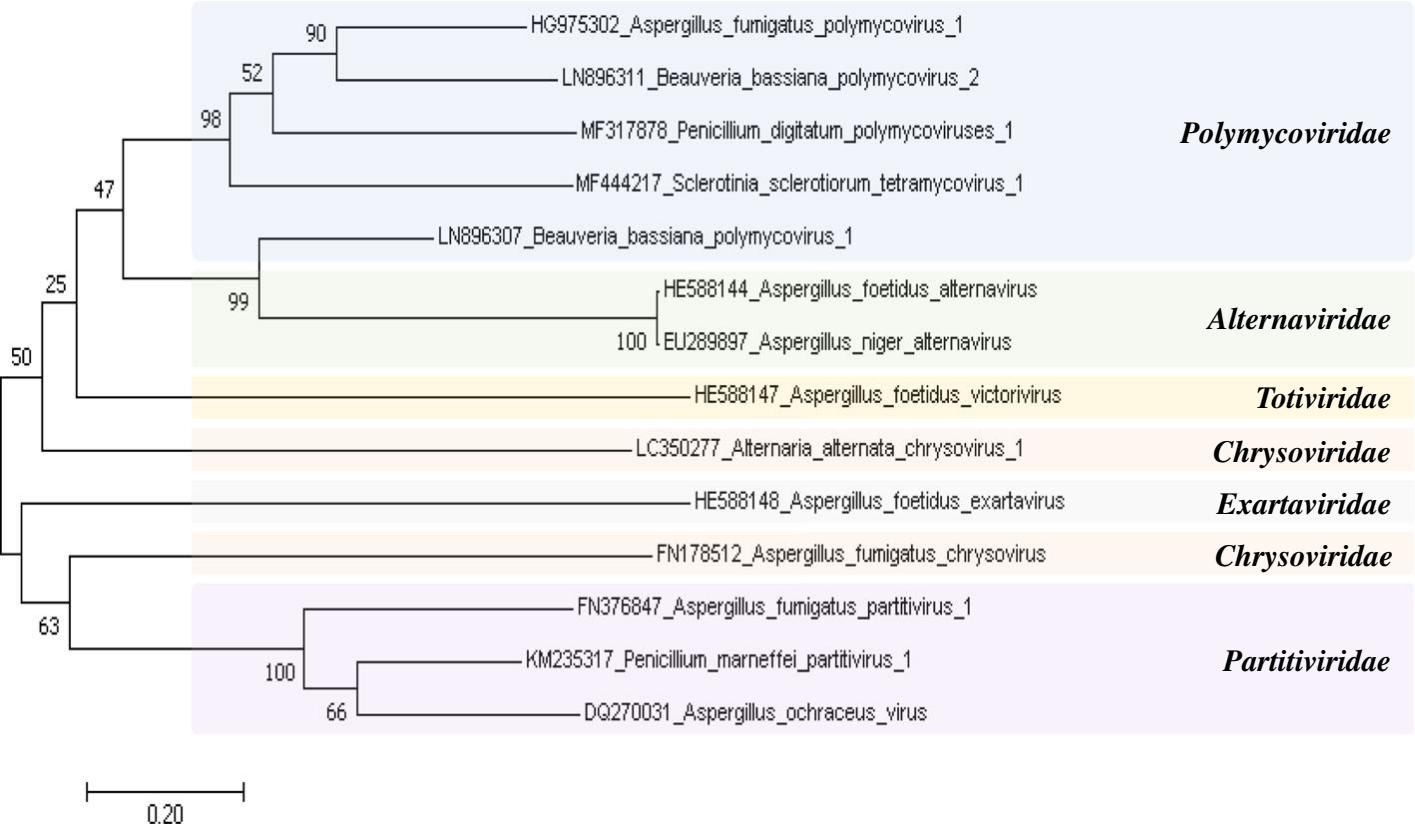
Evolutionary relationship based on the RdRp gene of the mycoviruses. The phylogenetic tree was constructed by using the Maximum Likelihood method based on the Kimura 2-parameter model. The analysis involved 14 nucleotide sequences of mycoviruses including polymycovirus. Evolutionary analyses were conducted in MEGA7. The bootstrap value was calculated as 1000 replicates

A phylogenetic tree was constructed to examine the phylogenetic relationships among the 14 mycoviruses, including AfuPmV-1. Polymycoviruses were grouped with AfuPmV-1. Polymycoviruses showed a relatively close relationship with alternaviruses (Fig. 5). To provide more information, the RdRP family for each sequence was examined from Pfam. AfuPmV-1 was assigned to RdRP_1 and all other polymycoviruses with similar codon patterns were assigned to the same RdRP_1 (pfam00680). Other mycoviruses infecting *Aspergillus* were classified as RdRP_1 or RdRP_4, although they were found in the same fungus (Table 5). This result suggests that the hypervirulent effects of AfuPmV-1 may be more affected by viral genome characteristics than by the effect of *Aspergillus* as a host.

**Table 5:** RdRP family as assigned by Pfam

No.	Accession no.	Mycovirus	Family by Pfam	E-value
1	FN376847	*Aspergillus fumigatus* partitivirus-1	RdRP_1	1e-06
2	FN178512	*Aspergillus fumigatus* chrysovirus	RdRP_4	1.8e-73
3	HG975302	*Aspergillus fumigatus* polymycovirus-1	RdRP_1	3.9e-05
4	HE588144	*Aspergillus foetidus* alternavirus	RdRP_4	3.6e-07
5	HE588147	*Aspergillus foetidus* victorivirus	RdRP_4	1.2e-105
6	HE588148	*Aspergillus foetidus* exartavirus	RdRP_1	4.4e-10
7	DQ270031	*Aspergillus ochraceus* partitivirus	RdRP_1	7.7e-12
8	EU289897	*Aspergillus niger* alternavirus	RdRP_4	3.8e-07
9	MF444217	*Sclerotinia sclerotiorum* tetramycovirus-1	RdRP_1	0.00017
10	MF317878	*Penicillium digitatum* polymycoviruses 1	RdRP_1	4.4e-07
11	LN896307	*Beauveria bassiana* polymycovirus 1	RdRP_1	2.5e-07
12	LN896311	*Beauveria bassiana* polymycovirus 2	no match	–
13	LC350277	*Alternaria alternata* chrysovirus 1	RdRP_4	7.7e-44
14	KM235317	*Penicillium marneffei* partitivirus-1	RdRP_1	4.1e-10

## Discussion

The mechanism underlying the hypervirulence effect of polymycoviruses has yet to be determined. However, experimental studies have demonstrated the existence of mycoviruses with mild hypervirulence effects, and other mycoviruses with similar sequences are continuously being discovered. The pathogenic effects of pathogenic fungi on the hosts may be increased by infection with mycoviruses that show hypervirulence effects. Therefore, it is necessary to determine the genetic characteristics of mycoviruses with hypervirulence effects. The results of the present study showed that AfuPmV-1 has a high GC content, and all of the strongly preferred codons (RSCU value ≥1.6) ended with either a C or G nucleotide. The distinctive codon usage pattern of AfuPmV-1 compared to other mycoviruses that infect *Aspergillus* spp. may be related to its hypervirulence effect. These characteristics did not appear in all mycoviruses with hypervirulent effects, but were shared by polymycoviruses. Nucleotide composition and codon usage patterns of polymycoviruses may be useful in predicting hypervirulent effects of unidentified mycoviruses.

## Conclusions

*Aspergillus* spp. are pathogenic fungi that cause various symptoms in humans. The hypervirulence effect of mycoviruses can increase the toxicity of *Aspergillus* spp. in human hosts, and thus increase the severity of symptoms. Here, AfuPmV-1 was shown to have distinct patterns in some codon usage indexes compared to other mycoviruses that infect *Aspergillus* spp. The distinctive codon usage pattern of AfuPmV-1 demonstrated in the present study indicated the need for monitoring of mycoviruses with similar characteristics. Research on mycoviruses has generally focused on their hypovirulence effects on fungi that infect plants. With the discovery of polymycoviruses, further research on the hypervirulence effects of mycoviruses is needed, particularly with regard to mechanisms of virulence control in mycoviruses, such as AfuPmV-1, which infects human pathogenic fungi.

## Abbreviations

AfuCV: *Aspergillus fumigatus* chrysovirus
AfuPmV-1: *Aspergillus fumigatus* polymycovirus-1
AfuPV-1: *Aspergillus fumigatus* partitivirus-1
AfuTmV-1: *Aspergillus fumigatus* tetramycovirus-1
BbPmV-1: Beauveria bassiana polymycovirus-1
CAI: Codon adaptation index
COA: Correspondence analysis
dsRNAs: Double-stranded RNAs
ENC: Effective number of codons
RdRP: RNA-dependent RNA polymerase
RSCU: Relative synonymous codon usage
SD: Standard deviation
